# Double Morphology: Tertiary Syphilis and Acquired Immunodeficiency Syndrome—A Rare Association

**DOI:** 10.1155/2017/3843174

**Published:** 2017-10-31

**Authors:** R. M. Ngwanya, B. Kakande, N. P. Khumalo

**Affiliations:** Groote Schuur Hospital and The University of Cape Town, Cape Town, South Africa

## Abstract

**Background:**

Human immunodeficiency virus (HIV) and* Treponema pallidum* coinfection is relatively common and accounts for about 25% of primary and secondary syphilis. Tertiary syphilis in HIV-uninfected and HIV-infected patients is vanishingly rare. This is most likely due to early treatment of cases of primary and secondary syphilis. There is rapid progression to tertiary syphilis in HIV-infected patients.

**Case Presentation:**

A 49-year-old woman diagnosed with HIV Type 1 infection and cluster of differentiation 4 (CD4) count of 482 presented with a four-week history of multiple crusted plaques, nodules, and ulcers on her face, arms, and abdomen. Her past history revealed red painful eyes six months prior to this presentation. She had generalized lymphadenopathy, no alopecia, and no palmar-plantar or mucosal lesions. There were no features suggestive of secondary syphilis. Neurological examination was normal. Her rapid plasma reagin test was positive to a titer of 64. She was treated with Penicillin G 20 mu IVI daily for 2 weeks.

**Conclusion:**

Penicillin remains the treatment of choice in syphilitic infected HIV negative and HIV-infected individuals. In neurosyphilis, the dose of Penicillin GIVI is 18–24 mu daily for 10–14 days. This case report demonstrates the importance of excluding syphilis in any HIV-infected patient.

## 1. Background

Human immunodeficiency virus and* Treponema pallidum* coinfection is relatively common and accounts for about 25% of primary and secondary syphilis [1]. Tertiary syphilis in the antibiotic era is rare and tertiary syphilis in human immunodeficiency virus- (HIV-) uninfected and HIV-infected patients is vanishingly rare. This is most likely due to the judicious use of antibiotics and early treatment of cases of primary and secondary syphilis. The standard of care for syphilis is penicillin. We present a 49-year-old female patient who had tertiary syphilis characterized by gummas and papillitis on a background of HIV coinfection.

## 2. Case Presentation

**Figure 1:**
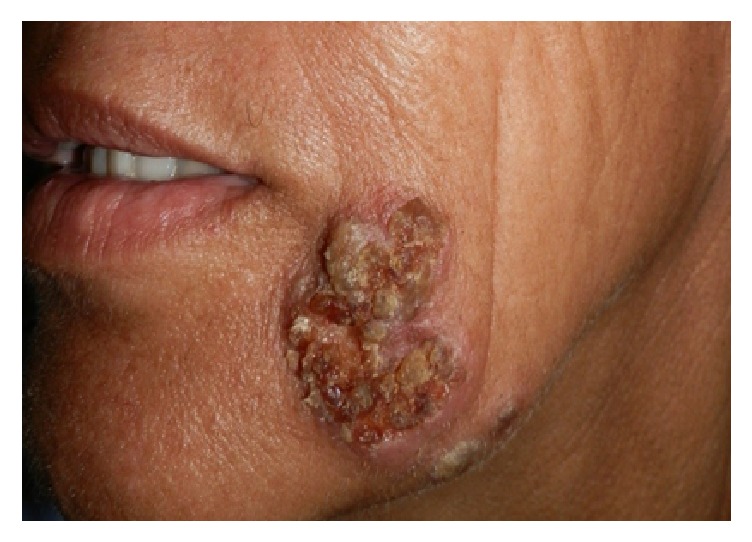
Plaque on chin before Penicillin G treatment.

**Figure 2:**
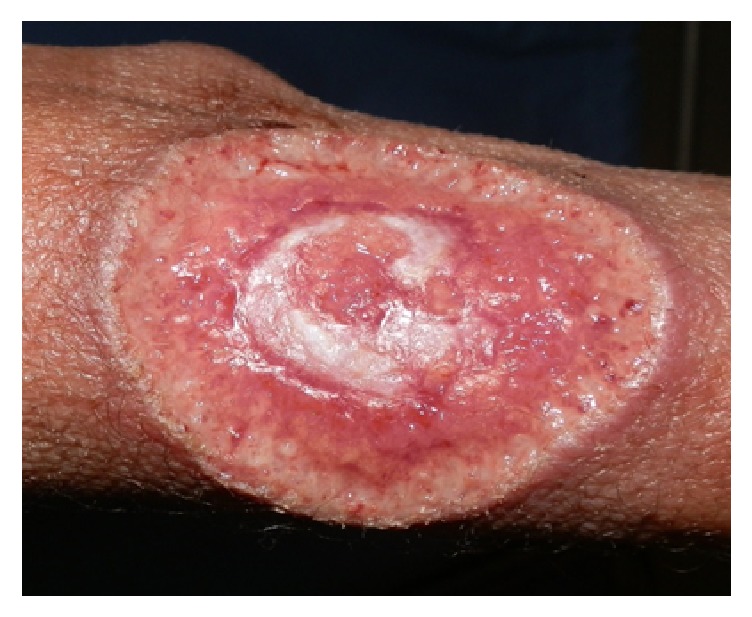
Ulcer on forearm before Penicillin G treatment.

A 49-year-old woman patient diagnosed with HIV Type 1 infection and cluster of differentiation 4 (CD4) count of 482 presented to the Dermatology Department at Groote Schuur Hospital, Cape Town, South Africa, with a four-week history of multiple crusted plaques, nodules, and ulcers on her face, arms, and abdomen (Figures 1 and 2). She was on antiretroviral therapy. Her past medical history revealed red painful eyes six months prior to this presentation. She did not seek medical care for her eye condition. She had generalized lymphadenopathy. no alopecia, no palmar-plantar, and no mucosal lesions or lesions of lues maligna. There were no features suggestive of secondary syphilis such as a papulosquamous nonitchy eruption on her body. Neurological examination was normal. Ophthalmological examination revealed unilateral acute papillitis. The physical examination showed a well looking female patient with crusted plaques and nodules on face and abdomen and an ulcer on her forearm, hence double morphology (Figures 1 and 2).

**Figure 3:**
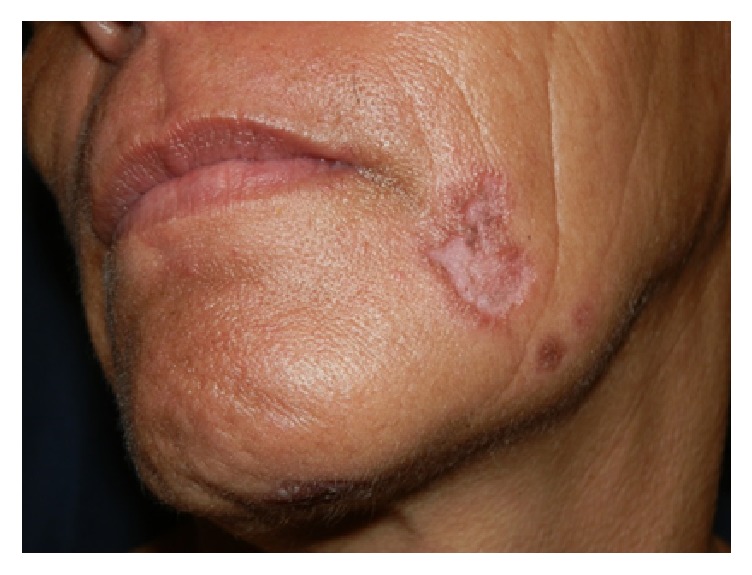
Plaque on chin after IVI Penicillin G treatment.

**Figure 4:**
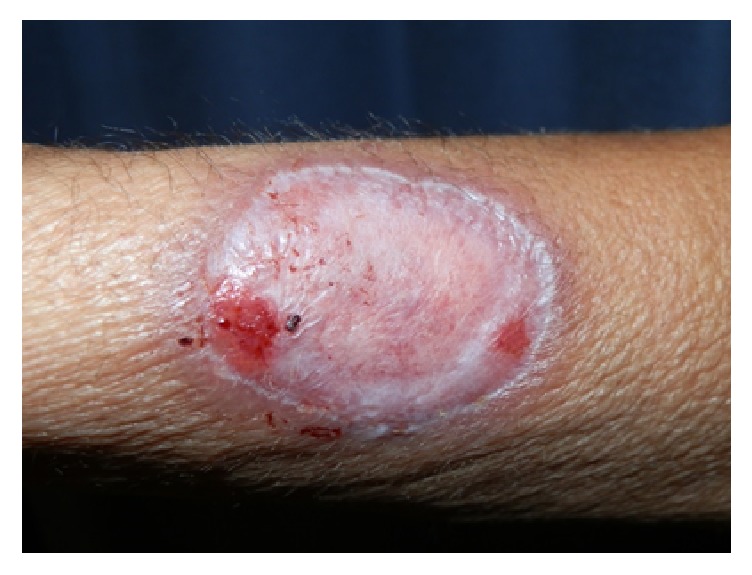
Ulcer on forearm after IVI Penicillin G treatment.

Our initial evaluation centered around lues maligna but this was excluded on the basis of the fact that lesions of lues maligna are usually multiple, well demarcated rupioid nodules and papules. A black eschar is sometimes observed on the lesions. Infections such as tuberculosis and deep fungal infections were also considered as atypical presentations are common in HIV infections. The patient was referred to an ophthalmologist and a diagnosis of papillitis was made. Rapid plasma reagin (RPR) test was positive (titer = 1 : 256, normal < 1 : 16). A diagnosis of tertiary syphilis with HIV coinfection was made. Tertiary syphilis diagnosis was made on the basis of the skin findings of gummas and papillitis, a manifestation of neurosyphilis. Lumbar puncture and skin biopsy were not done. She was treated with intravenous Penicillin G 5 MU IVI 6 hourly for 2 weeks. Healing occurred with atrophic scaring (Figures 3 and 4).

## 3. Discussion

Human immunodeficiency virus and* Treponema pallidum* coinfection is relatively common and accounts for about 25% of primary and secondary syphilis [1]. Tertiary syphilis in the antibiotic era is rare and tertiary syphilis in HIV-uninfected and HIV-infected patients is vanishingly rare. This is most likely due to the judicious use of antibiotics and early treatment of cases of primary and secondary syphilis. There is rapid progression to tertiary syphilis in HIV-infected patients, resulting in earlier onset of cardiovascular and neurologic sequelae [2]. Optic neuritis, uveitis, and other ocular manifestations of syphilis are common among HIV-infected patients [3]. Syphilis, a multisystem bacterial infection, is caused by the spirochete* Treponema pallidum*. It is characterized by three stages of active disease primary, secondary, and tertiary with latent periods between the secondary and tertiary stages. Gummatous mucocutaneous lesions of tertiary syphilis and advanced secondary syphilis can be seen simultaneously [4]. Left untreated patients with latent syphilis will develop tertiary syphilis. Tertiary syphilis usually is slowly progressive and without treatment will appear after many years characterized by the presence of neurosyphilis, cardiovascular syphilis, and gummas. The gummas occur in any organ but are seen more often in bone, mucous membrane, and the skin. Skin gummas which are by far very common are indurated, nodular, or ulcerative and spirochetes in these lesions are rare. Our patient presented with both the ulcers and nodules with plaques hence the double morphology. Our patient also presented with generalized lymphadenopathy. Lymphadenopathy is a usual finding in patients with both primary and secondary syphilis. It was not a surprising finding in our patient as this is a normal finding in HIV infection. Histopathological findings include a central area of coagulative necrosis, obliterative endarteritis, plasma cells, and epithelioid histiocytes. Papillitis and gummas, which were present in our patient, are manifestations of tertiary syphilis. Syphilis and HIV frequently coexist and this is not surprising [5]. The rate of coinfection has been noted to be on the rise in men having sex with men. Unusual and atypical forms of syphilis are usually observed in the setting of syphilis. In the presence of HIV infection syphilis can appear at any stage. The eye changes of syphilis can involve any structure of the eye with uveitis being the initial manifestation in patients who have acquired immunodeficiency syndrome [6]. Panuveitis is common in patients who are HIV infected [7]. Papillitis, also known as optic neuritis, is characterized by inflammation and deterioration of the optic disc. It is one of the many manifestations of neurosyphilis. Neurosyphilis is a serious complication of syphilis and can occur at any time during the course of syphilis. Syphilitic optic neuropathy has been described in seven patients who had syphilitic optic neuropathy on a background of HIV infection [8]. The presence of neurosyphilis should be looked for as this impacts on management. Diagnosis of syphilis is based on serological tests for both the non-*Treponema* and* Treponema* antibodies. It is important to actively test for syphilis in HIV-infected patients even when the initial test may be negative [9]. In our laboratory a titer of more than 1 : 16 is considered positive. Penicillin remains the treatment of choice in all forms of syphilis in both the HIV negative and HIV-infected individuals. In neurosyphilis the dose is Penicillin G intravenous (IVI) 18–24 mu daily for 10–14 days

She was treated with intravenous Penicillin G 5 MU IVI 6 hourly for 2 weeks. Healing occurred with atrophic scaring (Figures 3 and 4). That which occurs commonly must always be excluded even for rarer variants of that disease. This case report demonstrates the importance of excluding syphilis in any HIV-infected patient.

## Abbreviations

AIDS: Acquired immunodeficiency syndrome
HIV: Human immunodeficiency virus
CD4: Cluster of differentiation 4
RPR: Rapid plasma reagin
IVI: Intravenous.
